# Growth ranking of hybrid aspen genotypes and its linkage to leaf gas exchange

**DOI:** 10.1186/s12870-024-05104-6

**Published:** 2024-05-22

**Authors:** Ott Kangur, Reeno Sopp, Arvo Tullus, Priit Kupper, Eele Õunapuu-Pikas, Hardi Tullus, Reimo Lutter

**Affiliations:** 1 Chair of Silviculture and Forest Ecology, Institute of Forestry and Engineering, Estonian University of Life Sciences, Kreutzwaldi 5, Tartu, 51006 Estonia; 2 Department of Botany, Institute of Ecology and Earth Sciences, University of Tartu, Liivi 2, Tartu, 50409 Estonia

**Keywords:** Genotype ranking, Nitrogen, Populus, Water-use efficiency

## Abstract

**Background:**

Afforestation of non-forestland is a new measure by the European Union to enhance climate mitigation and biodiversity. Hybrid aspen (*Populus tremula* L. × *P. tremuloides* Michx.) is among the suitable tree species for afforestation to produce woody biomass. However, the best performing genotypic material for intensive biomass production and its physiological adaptation capacity is still unclear. We compared 22 hybrid aspen genotypes growth and leaf physiological characteristics (stomatal conductance, net photosynthesis, intrinsic water-use efficiency) according to their geographical north- or southward transfer (European *P. tremula* parent from 51° to 60° N and North American *P. tremuloides* parent from 45° to 54° N) to hemiboreal Estonia (58° N) in a completely randomized design progeny trial. We tested whether the growth ranking of genotypes of different geographical origin has changed from young (3-year-old) to mid-rotation age (13-year-old). The gas exchange parameters were measured in excised shoots in 2021 summer, which was characterised with warmer (+ 4 °C) and drier (17% precipitation from normal) June and July than the long-term average.

**Results:**

We found that the northward transfer of hybrid aspen genotypes resulted in a significant gain in growth (two-fold greater diameter at breast height) in comparison with the southward transfer. The early selection of genotypes was generally in good accordance with the middle-aged genotype ranking, while some of the northward transferred genotypes showed improved growth at the middle-age period in comparison with their ranking during the early phase. The genotypes of southward transfer demonstrated higher stomatal conductance, which resulted in higher net photosynthesis, and lower intrinsic water-use efficiency (iWUE) compared with northward transfer genotypes. However, higher photosynthesis did not translate into higher growth rate. The higher physiological activity of southern transferred genotypes was likely related to a better water supply of smaller and consequently more shaded trees under drought. Leaf nitrogen concentration did not have any significant relation with tree growth.

**Conclusions:**

We conclude that the final selection of hybrid aspen genotypes for commercial use should be done in 10–15 years after planting. Physiological traits acquired during periods of droughty conditions may not fully capture the growth potential. Nonetheless, we advocate for a broader integration of physiological measurements alongside traditional traits (such as height and diameter) in genotype field testing to facilitate the selection of climate-adapted planting material for resilient forests.

**Supplementary Information:**

The online version contains supplementary material available at 10.1186/s12870-024-05104-6.

## Introduction

New European forest and biodiversity strategy for 2030 envisages the planting of an additional three billion trees on non-forest lands to increase atmospheric CO_2_ uptake, produce woody biomass for future bioeconomies, and improve biodiversity [1]. In Northern Europe, the potential available land area for afforestation is around two million hectares; in Estonia, the forest cover is over 50% from the total land and the potential area for afforestation can be up to 0.3 million hectares [2].

At the same time, climate projections are predicting an increase in drought frequency during the vegetation period [3–5], which complicates the selection of the best climate-resilient plant material for future plantation establishment. The choice of the tree species and its genotypes adaptation to climate changes are crucial indicators to ensure the highest CO_2_ uptake and climate benefit [6, 7].

*Populus* spp. are considered as one of the most promising species for fast biomass production in the northern hemisphere [8, 9]. Among the suitable tree species for intensive biomass production [6], hybrid aspen (*Populus tremula* L. × *P. tremuloides* Michx.) has shown a potential to be more productive than native tree species of Northern Europe [10, 11]. Hybrid aspen can produce more than 20–25 m^3^ ha^− 1^ yr^− 1^ of stemwood at the end of the first 25-year rotation cycle for industrial woody biomass and renewable energy in short-rotation forestry systems [11–14], and at the same time preserve soil carbon pools [15] and increase biodiversity [16, 17] in comparison with agricultural land-use.

The ecosystem CO_2_ fixation and accumulation are mainly described with net production, which is mainly driven by resource supply. On the other hand, tree-level productivity is also controlled by genetic factors. Hybrid aspen planting material is produced through micropropagation [18]. The choice of clones for the local soil and environmental conditions is important to achieve the highest productivity and climate benefit through genetic improvement [9, 19]. The productivity of hybrid aspen plantations is mainly driven by site quality, however large genotypic variability of individual trees’ growth performance can be seen within the specific soil types as well [8, 10]. Moreover, the studies with *Populus* spp. are showing a great variability in genotypes growth performance in different environmental conditions [20–22]. This means that the adaptation of genetically different phenotypes for the given region must be tested at the regional level before large-scale plantation establishment.

Most of the comparison of *Populus spp* [21, 22]. and in particular hybrid aspen [20, 22] genotypes performance is carried out at the early growth phase (< 5 year). However, the early genotype selection for the commercial use might not describe their potential growth [13, 20, 23]. The stability of genotypes to maintain their early growth advantage over slower-growers depends on their physiological adaptation capacity to cope with extreme climatic events such as drought [9, 24]. In most cases, the early growth of the best-performing hybrid aspen genotypes links well with the latter-stage growth ranking [11, 13, 23], nonetheless the final choice of the best genotypes for wider commercial use is recommended at later development stage to obtain the growth potential in combination with the resistance to abiotic and biotic disturbances [13].

Hybrid aspen growth is under strong genotypic control [20, 25], but the question remains about the physiological mechanism of why some genotypes can achieve higher production rates in comparison with slower-growing genotypes under the same environmental conditions. The present genotype selection is based on the traditional phenological parameters such as height or diameter growth. However, the mechanistic understanding of trees’ physiological responses and its integration to future tree breeding programmes helps to select the best climate-adapted genotypes and establish resilient forests under the climate changes. So far, the knowledge of genotypes physiological parameters comparison and linkage with growth is poorly studied in field conditions in Northern Europe.

*Populus* spp. is characterized by high demand for water to maintain high production [26]. One possibility to improve forests’ adaptability to climate changes caused by stress factors is to consider genotypic control on physiological traits like water-use efficiency (WUE) [24, 27, 28]. WUE describes the amount of water lost during CO_2_ fixation in photosynthesis or per unit of dry biomass produced [29]. WUE is both under genetic and environmental control [9, 30, 31]. It has been shown in many studies that WUE is under genetic control also for *Populus* spp [9, 28, 32, 33]. However, a large controversy exists about whether WUE is linked with tree growth parameters. Aleta et al. [34] found that most water-use-efficient *Juglans regia* L. families exhibited less height growth and smaller diameter at breast height. Those were the genotypes that originated from drought-prone areas. Verlinden et al. [33] found no relationship between WUE and tree growth parameters in six poplar genotypes. However, González de Andrés et al. [35] demonstrated a positive connection between WUE and basal area increment for *Fagus sylvatica* L., while none for *Pinus sylvestris* L. To the best of our knowledge, there is only one study in the Northern Europe region that aimed to compare tree physiological parameters among hybrid aspen genotypes in field conditions at the early growth stage [22]. The study by Yu [22] did not find a relationship between gas exchange parameters and tree growth traits; however, transpiration rate was linked with leaf nitrogen (N) content, similar to other studies with *Populus* spp [33].

We compared the growth potential and leaf gas exchange parameters (converted also to WUE) of 22 hybrid aspen genotypes representing four geographical origins in hemiboreal Estonia. We hypothesize that: (1) the ranking of hybrid aspen genotypes by geographical origin has not changed from the young to middle-age period; (2) Net photosynthesis and leaf nitrogen are coupled with tree growth among the genotypes.

## Materials and methods

### Study site

The study was carried out in a progeny trial of hybrid aspen (*Populus tremula* × *P. tremuloides*) in south-eastern Estonia (58° 17′ 10″ N, 27° 17′ 18″ E). The trial was established with 1-year-old containerized plants in the spring of 2009 on former agricultural soil. The trial was planted with a completely randomized uniform design where 22 genotypes are presented in three replications. The initial spacing was 1 × 2 m where 16 individual trees from the same clone were planted in 4 × 4 arrangement per replication. In order to reduce light competition and avoid self-thinning, two harvests have been carried out at the trial site. Half of the trees were harvested systematically (every second row) after the fifth growth year in winter of 2013/14, retaining eight trees per replication. After the seventh growth year in winter 2015/16, another half of the remaining trees were harvested by retaining the highest four trees per replication. The data collection for the present study was first done at the 3rd growing season in 2011 (growth parameters), and the second time at the 13th growing season in 2021 (growth and physiological parameters).

The trial site is characterized by a flat surface and homogeneous soil conditions, referring to fertile *Eutric Glossic Retisol* with a sandy loam soil texture [36]. *Eutric Glossic Retisol* is the basis for *Oxalis* forest site type [37] and is the most widespread soil type of former agricultural soils in southern Estonia for afforestation with hybrid aspen plantations. Mechanical weed control was carried out at the establishment phase to reduce trees’ competition with understorey vegetation. The trial was fenced to avoid game damage.

**Fig. 1:**
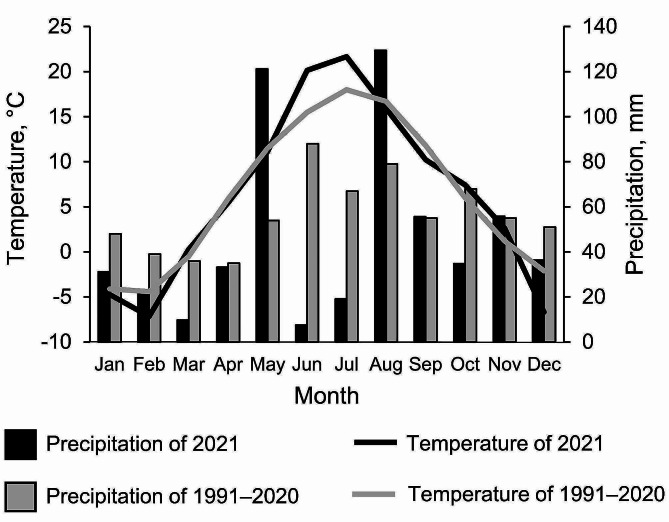
Temperature and precipitation of 2021 in comparison with the long-term climate normal values (Estonian Environment Agency)

The annual average temperature in sampling year 2021 was 6.6 °C and the sum of precipitation was 556 mm according to the nearest weather station (∼5 km) to the site (http://fahm.ut.ee/). The long-term climate normal (1991–2020) reports the average annual temperature of 6.3 °C and precipitation of 675 mm to south-eastern Estonia (Estonian Environment Agency). The sampling year 2021 is characterized with warmer and drier June and July where only 17% of precipitation was received in comparison with the long-term average (Fig. 1). The average temperature in 2021 June and July was 21 °C, which is 4 °C higher than the long-term temperature for June and July (Fig. 1).

### Origin of genotypes

The progeny trial aimed to compare different hybrid aspen (*Populus tremula* × *P. tremuloides*) clones and full-sib families from different geographic locations of their native European aspen (*P. tremula*) parent. In total, 22 different hybrid aspen progenies were tested according to their *P. tremula* latitudinal origin (a detailed source of genotypes is presented in Supplementary 1; Table S1): seven clones from Latvia (56° 06′ to 57° 10′ N); five clones from Sweden (55° 53′ to 57° 31′ N); four clones from Finland (60° 22′ N); and 6 full-sib families from Germany (51° 16′ to 52° 16′) [25]. The origin of *P. tremuloides* parent is from the USA and Canada (45° to 50° N in the Great Lakes region, 44° N New Hampshire, 45° 17′ Ontario, and 54° 06′ British Columbia), but less documented than for *P. tremula* [25]. The studied genotypes are crossed under the hybrid aspen breeding programs of the origin countries and assessed as among the best growers at their breeding location. German and Latvian plants were pre-grown at the nurseries of the origin countries, whereas Swedish and Finnish plants were pre-grown in Estonia. All the studied genotypes are deposited in 12 replications in the Agali genotypes trial (58°19′40″N, 26°33′16″E). Information about the genotypes’ origin and locations in the trial are stored in the non-public database of forestry experiments at the Estonian University of Life Sciences (formis.emu.ee).

### Growth parameters

Tree height (H, m) was recorded with a Vertex IV tool (Haglöf Sweden AB), and diameter at breast height (DBH, cm) with forest caliper at the end of the growing season. H and DBH were used to calculate stem volume (V, dm^3^), based on allometric equation developed for hybrid aspen [38]:

$$\begin{gathered} V{\text{ }} = {\text{ }}0.03186{\text{ }} \times {\text{ }}DB{H^2} \times {\text{ }}H{\text{ }} + {\text{ }}0.43{\text{ }} \times {\text{ }}H{\text{ }} + \hfill \\\,\,\,\,\,\,\,\,\,\,\,\,\,\,\,\,\,\,\,\,\,\,{\text{ }}0.0551{\text{ }} \times {\text{ }}DB{H^2} - {\text{ }}0.4148{\text{ }} \times {\text{ }}DBH \hfill \\ \end{gathered}$$

Stem mass (M, kg) was converted from the V estimate by applying wood density value for each studied genotype from model tree sampling at the age of 5 years. The wood density was obtained from the stem discs from 1.3 m height as fresh volume to oven-dried weight without bark. The current annual stem mass increment (CAIM) was calculated by subtracting the M at the end of year 2020 from the M at the end of year 2021 for each tree. We also calculated yearly relative growth (RG, kg kg^− 1^ yr^− 1^) by dividing CAIM with the tree M_2020_, and relative growth rate (RGR, g kg^− 1^ day^− 1^) by dividing RG with vegetation period length in days and, in turn, multiplying that with 1000 to depict the result in grams [39]. The length of the vegetation period is number of days from bud-burst (leaves emerge from the bud about 5 mm) to defoliation of all the leaves at age 6 years based on visual observations [25].

Genotypes by individual trees were ranked according to their heights after the 3rd and after the 13th growing seasons and merged into their origin groups. The change of the ranking was converted to a relative scale (%) to assess the stability of genotypes and their origins over the 10-year period (at the end of the growing seasons in 2011 and 2021).

### Measurements and calculation of physiological parameters

The gas exchange experiment followed the experimental setup used by Rohula et al. [40] and Kupper et al. [41]. The measurements were conducted on excised shoots of the study plants in the laboratory from 22nd to 30th July 2021. The sample shoots (two per genotype) were collected on the previous evening of the measurement day. Sample branches were cut from the upper third of the tree’s canopy with telescope clippers, and the sample shoot was cut from the branch with a razor blade under deionized (Direct-Q3 UV water purification system; Millipore SAS, Molsheim, France) and freshly degassed water (T‐04‐125 ultrasonic‐vacuum degasser; Terriss Consolidated Industries, Asbury Park, NJ, USA) and inserted into a water‐filled plastic container. The shoots were transported to the laboratory, where their cut ends were recut under water (1.5 h after the first cutting). The submerged leaves of the cut shoots, demonstrating clear signs of water infiltration, were removed during the recutting. The distance between the previous and new cut was at least three internodes. The recut shoots (20–40 cm tall) were inserted into 100 ml plastic flasks filled with deionised and degassed water and placed in shaded outside conditions to rehydrate overnight. The next morning shoots were placed under an artificial light source (SON-T AGRO high-pressure sodium lamps, 400 W; Philips, Eindhoven, The Netherlands) at constant light (800 µmol m^− 2^ s^− 1^) and controlled temperature (21.7–22.5 °C) conditions of the lab.

Leaf gas exchange was measured with a portable gas exchange system (CIRAS-2; PP‐Systems, Amesbury, MA, USA) on two fully developed leaves per shoot. The net photosynthesis (*P*_n_; µmol m^− 2^ s^− 1^), daytime stomatal conductance (*g*_day_; mmol m^− 2^ s^− 1^), and transpiration rate (*E*; mmol m^− 2^ s^− 1^) were measured from 8:00 to 13:00 hr. The gas exchange measurements were carried out at constant irradiance (800 µmol m^− 2^ s^− 1^), cuvette temperature (24°C), cuvette CO_2_ concentration (400–410 ppm), and ambient air humidity (60–65%). Bulk leaf water potential (Ψ_L_; MPa) was determined by the balancing pressure technique [42] with a Scholander‐type pressure chamber (Model 1000 Pressure Chamber Instrument; PMS Instrument Company, Albany, OR, USA) on all investigated shoots (on two to three leaves per shoot) immediately after gas exchange measurements. Intrinsic water-use efficiency (iWUE; µmol mol^− 1^) was calculated as *P*_n_/*g*_day_. Whole shoot (leaves + branch) hydraulic conductance (*K*_shoot_; mmol m^− 2^ s^− 1^ MPa^− 1^) was estimated by the evaporative flux method [43] and calculated according to the Ohm’ Law analogy:

$${K_{shoot}} = E \div {\text{ }}\left( {{\Psi _W} - {\text{ }}{\Psi _L}} \right)$$

Where Ψ_W_ is the water potential of distilled water in the flask, which is ∼0. Therefore, the formula can be simplified as:

$${K_{shoot}} = E \div {\text{ }}\left( { - {\text{ }}{\Psi _L}} \right)$$

Since *E* is expressed per unit leaf area and the measurements were done under steady-state conditions, the values of *K*_shoot_ are scaled by leaf area and standardized for the dynamic viscosity of water.

Leaf nitrogen (N, %) concentration was determined with the Kjeldahl method, using a Kjeltec Auto 1030 Analyzer (Fross Tecator Sweden AB) in the Laboratory of Plant Biochemistry and the Laboratory of the Department of Soil Sciences and Agrochemistry at the Estonian University of Life Sciences.

### Statistical analysis

Mixed model ANOVA was used to estimate the effect of the origin groups on all the parameters. For the change of growth ranking, all individual trees were ranked at the ages of 3 and 13 according to their height, including only the trees that were present at both age periods. For each tree, the ranking at the age of 13 was subtracted from the ranking at the age of 3, i.e. positive change means the improved ranking and negative change means the drop in the ranking. The effect of the origin group on the change of the ranking was tested with a linear mixed model where replication was treated as a random factor. For physiological parameters, date of measurement, tree specimen, and genotype were treated as random factors, for growth parameters only genotype was treated as random factor.

Mixed model ANOVA was also used to estimate the effect of genotypes on physiological parameters, whereas date of measurement and tree specimen were treated as random factors, and on growth ranking, whereas replication was treated as a random factor. One-way ANOVA was used to estimate the effect of genotypes on growth parameters that were measured on the same specimen on which the physiological parameters were measured. Post hoc comparisons of group means were calculated with Tukey’s HSD method. We also applied an analysis of covariance (ANCOVA) to assess the co-effect of the origin group and various continuous variables on *P*_n_ and iWUE. The normality of the variables was tested with the Kolomogorov-Smirnov test. Levene’s test was applied to assess the homogeneity of variances. All statistical tests were considered significant when *P* < 0.05. All statistical analyses were performed in the R software version 4.2.2 [44].

## Results

### Growth parameters

**Fig. 2:**
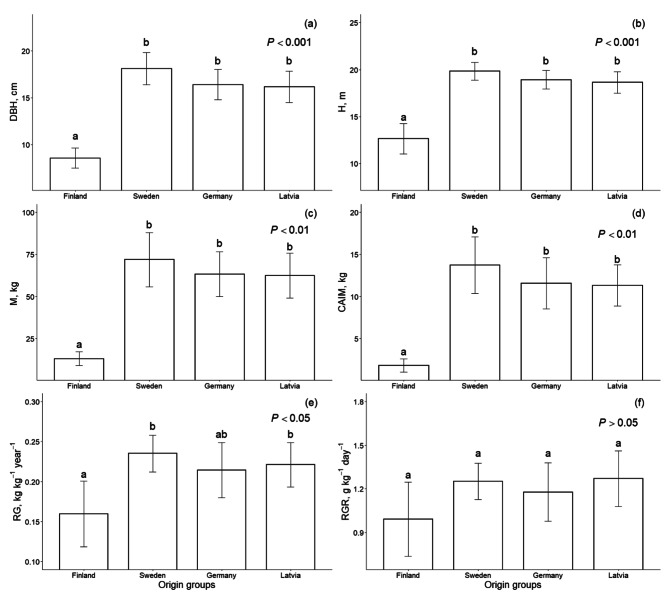
Diameter at breast height (DBH) **(a)**, height (H) **(b)**, stem mass (M) **(c)**, current annual stem mass increment (CAIM) **(d)** yearly relative growth (RG) **(e)** and relative growth rate (RGR) **(f)** of hybrid aspen clones from four different origin groups at the age of 13 years. Different letters denote significant differences in mean values between the groups

Tree growth parameters such as DBH, H, M, and CAIM significantly differed among origin groups at the end of the 13th growing season. DBH, H, M, and CAIM were the lowest for the Finland-origin group, whereas mean values did not differ among the other origin groups (Fig. 2a, b and c, and 2d). Mean DBH was approximately 50%, H was 35%, M was 80% and CAIM was 85% smaller for the Finland-origin group than other groups. RG also differed between origin groups (*P* < 0.01), being the lowest for Finland-, and highest for Sweden- and Latvia-origin groups. German-origin group was in middle and did not significantly differ from any other origin group (Fig. 2e). There was a similar trend for RGR, however it did not significantly differ between the origin groups (Fig. 2f).

**Fig. 3:**
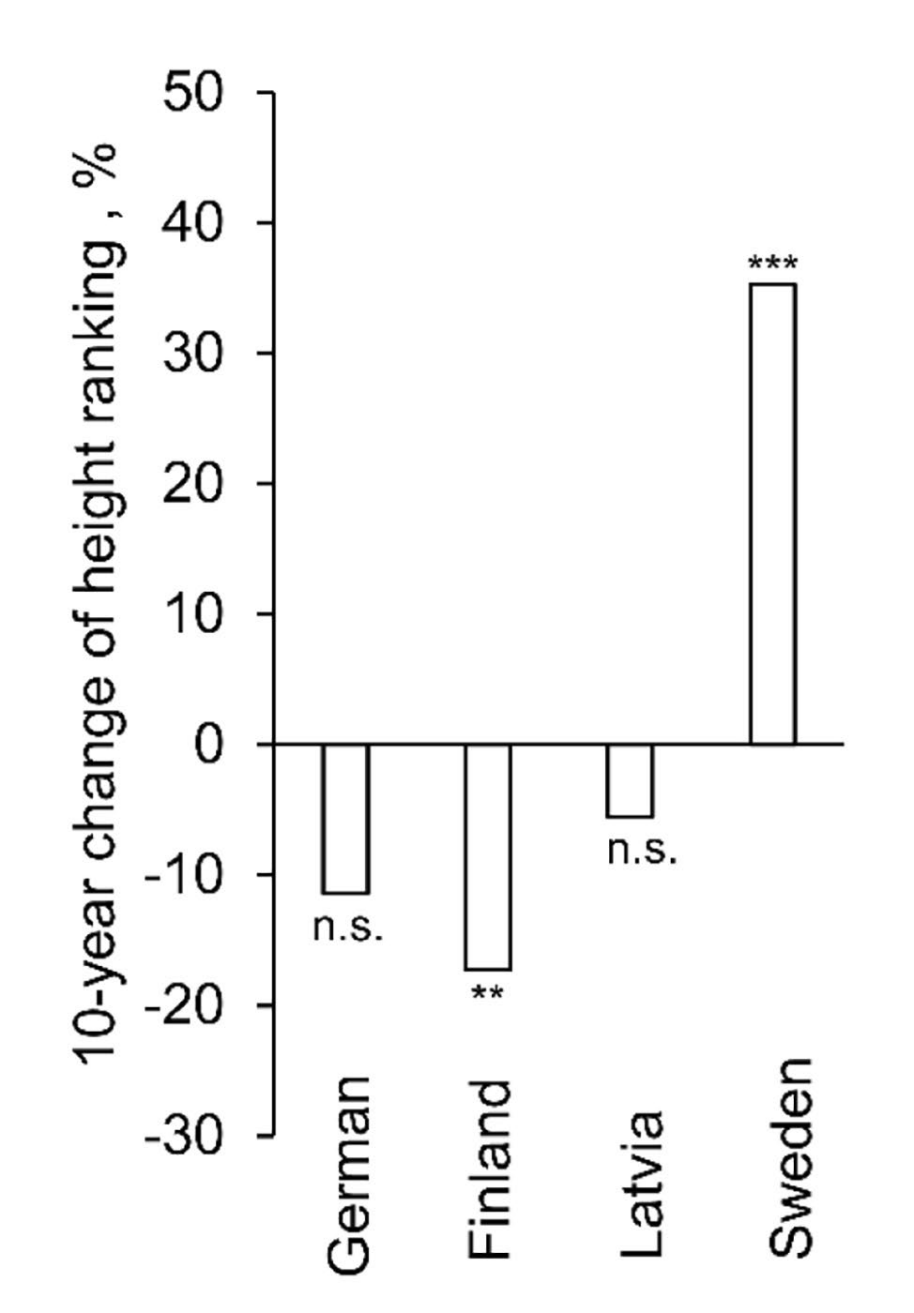
Ten-year (3- to 13-year-old) change of relative height ranking among the genotypes` geographical origins. The significance of the mean change of ranking inside the origin group (i.e. difference from zero): *** *P* < 0.001, ** *P* < 0.01, * *P* < 0.05, n.s. - not significant

Ten genotypes out of 22 exhibited a significant change in growth ranking after 10 years, with five of them significantly higher and five of them with significantly lower ranking (Supplementary 1; Figure S1). Only the German-origin group contained genotypes that did not exhibit any significant change in the ranking. The Latvia-origin group stood out with having genotypes both with significant increase and decrease in ranking, however, that led to having no change in ranking for the Latvia-origin group in general. Only the Finland-origin group demonstrated a significant decrease (*P* < 0.01) and the Sweden-origin group demonstrated significant increase (*P* < 0.001) in ranking over the 10 years from the age of 3 to 13 (Fig. 3). Pearson correlation coefficient showed a significant relationship (0.6; <0.001) between the two sampling occasions over all studied genotypes.

### Physiological parameters

**Fig. 4:**
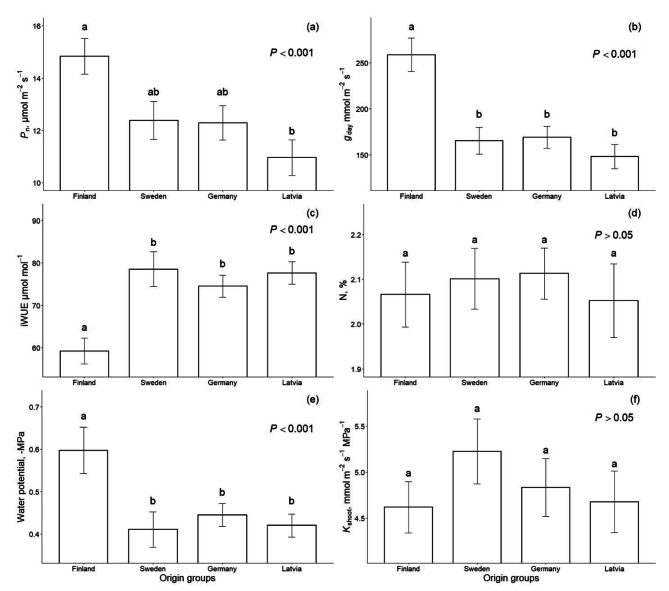
Mean net photosynthetic rate (*P*_n_) **(a)**, leaf stomatal conductance (*g*_day_) **(b)**, intrinsic water use efficiency (iWUE) **(c)**, leaf nitrogen content (N) **(d)**, leaf water potential **(e)** and shoot hydraulic conductance (*K*_shoot_) **(f)** of hybrid aspen clones from four different origin groups measured between 22nd and 30th July 2022. Different letters denote significant differences in mean values between the groups

**Fig. 5:**
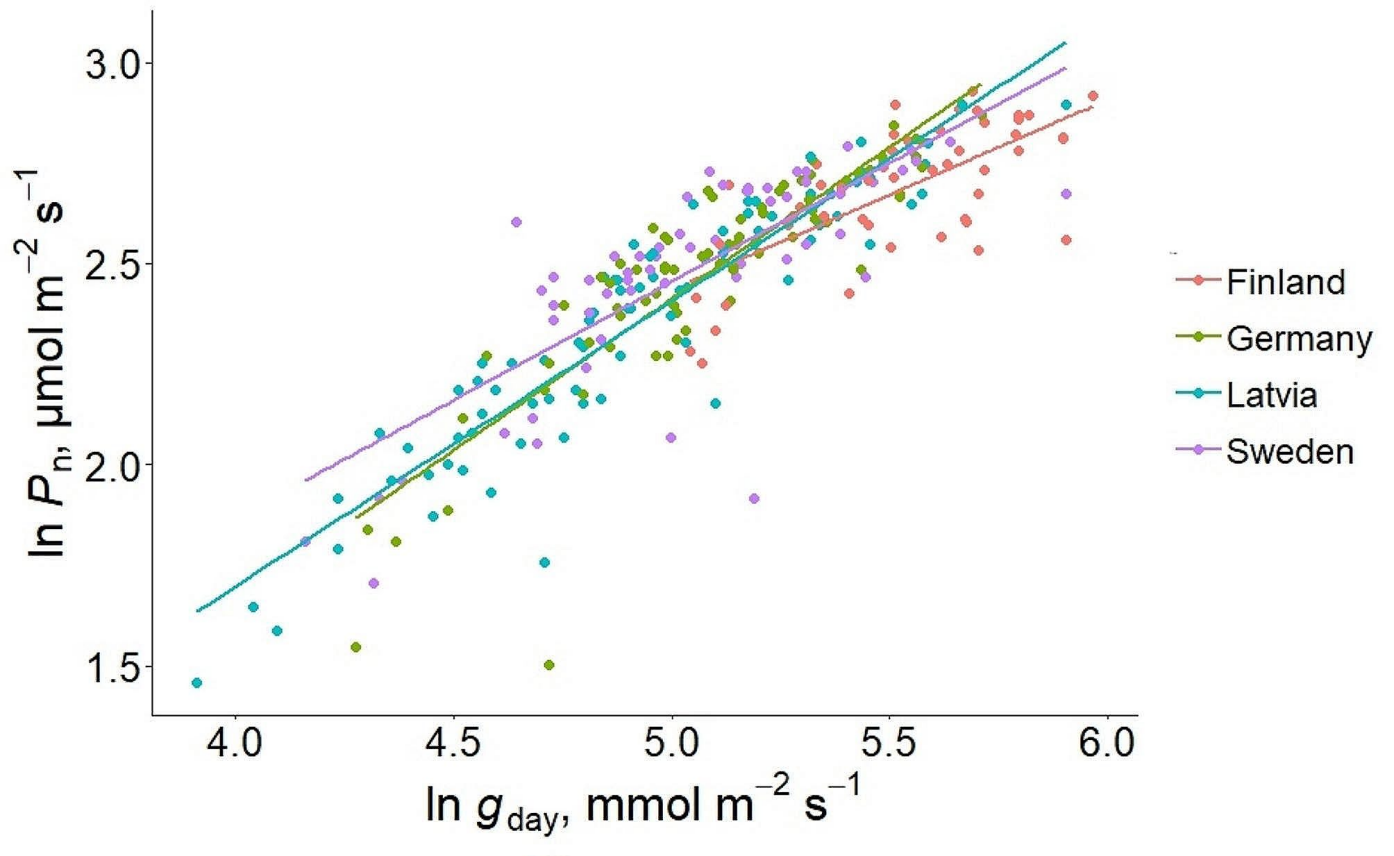
The effect of daytime stomatal conductance (*g*_day_) and the origin group on net photosynthetic rate (*P*_n_) on hybrid aspen origins. Both, *g*_day_ and *P*_n_ values are on a logarithmic scale. Model *R*^2^ and model fit line equation values for the origin groups were as follows, respectively: Finland − 0.49, *P*_n_ = 0.47 × *g*_day_ + 0.08; Germany − 0.75, *P*_n_ = 0.75 × *g*_day_ − 1.36; Latvia − 0.86, *P*_n_ = 0.71 × *g*_day_ − 1.15; Sweden − 0.61, *P*_n_ = 0.59 × *g*_day_ − 0.49, and *P* < 0.001 for all the origin groups. Different colors denote different origin groups

Mean *P*_n_ was the highest in Finland- and the lowest in the Latvia-origin group (*P* < 0.01), being approximately 25% lower than for Finland. *P*_n_ in Sweden- and German-origin groups did not differ from either of the first origin groups (Fig. 4). Mean *P*_n_ also differed among genotypes (*P* < 0.001), being approximately 45% lower for the lowest performing genotype (Latvia6) than the highest performing genotype (Finland4). Within origin groups, *P*_n_ differed between genotypes only in the Latvia-origin group (*P* < 0.001). In other origin groups, we did not observe a significant difference (*P* > 0.05) between genotypes. There was also a coeffect of *g*_day_ and the origin group on *P*_n_. The respective model described 71% (*P* < 0.001) of the variance in *P*_n_ (Fig. 5).

*g*_day_ significantly differed between the Finland- and other origin groups (*P* < 0.001), being the highest for the Finland-origin group, with a mean value of 259 mmol m^− 2^ s^− 1^. *g*_day_ did not differ significantly between other origin groups, with mean values ranging from 148 to 169 mmol m^− 2^ s^− 1^ (Fig. 4b). Within origin groups *g*_day_ differed between genotypes in the Latvia- (*P* < 0.001) and the German-origin groups (*P* > 0.05). Within other origin groups, we did not observe significant differences (*P* > 0.05) between genotypes.

**Fig. 6:**
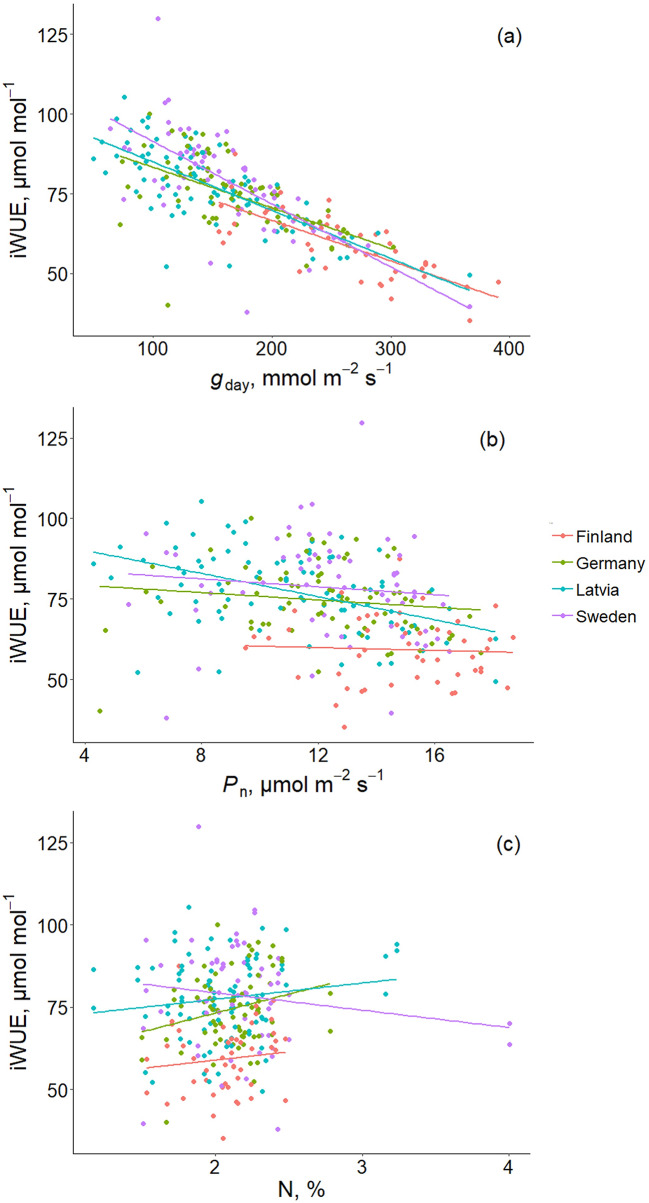
The effect of daytime stomatal conductance (*g*_day_) **(a)**, net photosynthetic rate (*P*_n_) **(b)**, and leaf nitrogen content (N) **(c)** on intrinsic water use efficiency (iWUE) on hybrid aspen origins. Model *R*^2^ and model fit line equation for the origin groups were as follows, respectively: **(a)** Finland − 0.58, iWUE = − 0.13 × *g*_day_ + 92.08; Germany − 0.34, iWUE = − 0.13 × *g*_day_ + 96.05; Latvia − 0.55, iWUE = − 0.15 × *g*_day_ + 100.06; Sweden − 0.50, iWUE = − 0.20 × *g*_day_ + 111.06, and *P* < 0.001 for all the origin groups; **(b)** Latvia − 0.21, iWUE = − 1.80 × *P*_n_ + 97.34, and *P* < 0.001; Finland, Germany, Sweden - nonsignificant, *P* > 0.05 (*R*^2^ and fit line equation not shown) **(c)** Germany − 0.06, iWUE = 11.47 × *N* + 50.31, and *P* < 0.05; Finland, Latvia, Sweden - nonsignificant, *P* > 0.05 (*R*^2^ and fit line equation not shown). Different colors denote different origin groups

iWUE differed between origin groups (*P* < 0.001), being higher for Sweden-, German-, and Latvia-origin groups and the lowest for the Finland-origin group (Fig. 4c). iWUE did not significantly differ (*P* > 0.05) between genotypes within any of the origin groups. The variation in iWUE was best described by the variation in *g*_day_ (Fig. 6a and b). Leaf N content did not affect iWUE (Fig. 6c). Moreover, leaf N content did not significantly differ between the origin groups (Fig. 4d). Water potential was the most negative in the Finland-origin group with a mean value of -0.60 MPa, which significantly differed (*P* < 0.001) from water potential values of other origin groups, with mean values between − 0.46 and − 0.42 MPa (Fig. 4e). Shoot hydraulic conductance did not differ between the origin groups (Fig. 4f).

## Discussion

### Growth

At the middle-age development phase (13-year-old), hybrid aspen genotypes originating from southern latitudes showed almost 3-fold higher growth potential (M and CAIM) in comparison with the northern origin. Our results indicate that the potentially increased risk of frost damage, which may accompany the northern transfer of *Populus* spp [45] was not reflected by the growth rate of hybrid aspen during the 13-yr period even in the case of genotypes originating from Germany, representing the longest transfer (by about 7 degrees of latitude) and late defoliation [25]. Such difference in growth parameters between southern and northern origin genotypes indicates that the establishment of new commercial plantations with southern origin genotypes could significantly improve the climate benefit for the European Union Forest strategy for 2030 [1]. For example, so far in Estonia, the large-scale commercial-level hybrid aspen plantation establishment is done only by using the genotypes from northern origin (Finland) [10].

In general, we found that the selection of genotypes based on their height growth at the early development phase (3 years) is in accordance with their latter selection (13 years). However, for some geographical origins such as Sweden, the early selection might underestimate their real growth potential as those genotypes improved their ranking by about 35%. And oppositely, northern genotypes lost their ranking in the latter survey. Our results are in good agreement with previous study about the hybrid aspen clones ranking, which suggested that the optimal period for the final selection should be between 10 and 15 years [13].

Our long-term experiment also demonstrates that genotypes originating from south (Sweden, Latvia, and Germany) exhibited similar growth rates even when grown in relatively mixed stands. This means that when cultivating these genotypes together and avoiding large areas of monoclonal plantations, representatives of any group are not significantly dominating over other genotypes or exhibit inferior growth compared to other genotypes. Therefore, the stand should maintain relatively uniform in growth. The advantage of cultivating genotypes of different geographical origins together could lie in the fact that genetically more diverse communities are generally more resistant to various pathogens and pests outbreaks [46–48].

### Effect of genotype origin group on physiological parameters

Physiological parameters were quite variable between the genotype origin groups. Similarly, with growth parameters, there was a clear pattern where the mean values of gas exchange parameters of the Finland - the northernmost - origin group differed from mean values of other origin groups. The Finland-origin group demonstrated the highest *P*_n_, which coincides with results from many other common garden experiments conducted on different poplar species [49–51]. A study on silver birch conducted in Finland demonstrated a similar trend, but the relationship was not very clear [52]. Our findings suggest that *g*_day_ was the major driver of the variation in *P*_n_: Finland-origin group demonstrated also the highest *g*_day_ values, and *P*_n_ and *g*_day_ correlated very well with each other. That is consistent with other similar studies [49, 51]. However, most of these studies additionally showed that higher *P*_n_ in northern provenances is attributable to higher photosynthetic capacity, which is usually expressed as leaf nitrogen (N) concentration as they are highly correlated [33, 53]. In our study this does not seem to be the case, as N did not significantly differ between the origin groups, indicating a high N supply from soil to trees. Therefore, we believe that in this study, which was conducted in relatively dry period compared to long-term precipitation trends, the variation of *g*_day_ was the main driver of differences in *P*_n_ among origin groups in hybrid aspen.

Despite demonstrating the highest *P*_n_ that in theory may lead to higher iWUE, the Finland-origin group demonstrated the lowest iWUE under a severe drought period, which is a result of almost twofold higher *g*_day_ compared to other origin groups. On the one hand, higher *P*_n_ and *g*_day_ in the Finland-origin group could be explained by the need to assimilate more CO_2_ to compensate for shorter vegetation periods in northern habitats [25, 50]. On the other hand, lower *g*_day_ and higher iWUE in southern origin groups could be explained by their stronger stomatal control, which is crucial for preventing cavitation in xylem vessels [54], which in turn is more likely to occur in areas with more intensive irradiance and higher temperatures. A study with silver birch reported similar patterns for *g*_day_ and iWUE related to the provenance of origin groups [52]. Studies with poplar species conducted in the USA and/or Canada have demonstrated slightly different patterns: *g*_day_ also increases with latitude but no trend in iWUE values [49, 51], or iWUE increases with latitude but no trend in *g*_day_ values [50].

Another explanation for why *g*_day_ and *P*_n_ varied between the origin groups could be a different reaction of different-sized trees to the relatively droughty conditions arising from limited precipitation. Southern clones were bigger and therefore, their canopies probably experienced higher vapor pressure deficit (VPD) values (exposure to higher irradiance and temperatures, and wind). As the shoot hydraulic conductance did not differ between the origin groups, the water supply to leaves was likely similar among the origin groups. Therefore, the differences in mean *g*_day_ and iWUE between the origin groups were not only determined by the genotypes’ origin per se, but also by the environmental conditions in this experiment. Most of the physiological parameters measured in this study expectedly varied between genotypes when the origin group was removed from the analysis. However, when comparing genotypes within single origin groups, the parameters usually did not differ between genotypes, e.g. within the Finland- and Sweden-origin group, none of the physiological parameters differed between genotypes. In that sense, the Latvia-origin group was the most diverse with *P*_n_ and *g*_day_ being different between genotypes. Small variations in physiological parameters within origin groups could be a result of relatively small genetic variation within respective origin groups.

### Relationship between physiological and growth parameters

Higher *P*_n_ demonstrated by the Finland-origin group in the 2021 growing season does not reflect in any of the growth characteristics: neither current year (CAIM, RG, RGR) nor total growth (H, DBH, M). That could be explained by the fact that trees in the Finland-origin group were significantly smaller compared to other origin groups already at the beginning of the 2021 growing season. Finland clones with smaller total leaf area just lacked the capacity to assimilate as much carbon as bigger trees from southern origin groups. Nevertheless, one could have assumed that higher *P*_n_ would have resulted in higher RGR as the latter is not directly related to tree size; however, that was not the case. The explanation for this could be that physiological measurements were done under controlled conditions, where all the clones experienced identical light conditions, whereas, on the study site, Finland clones were at least partially shaded by genotypes from other origin groups and, therefore, less prone to drought compared to clones form other origin groups. Another reason why southern genotypes demonstrated higher growth despite their lower *P*_n_ could be their longer growing season. A study on the same genotypes revealed that Latvia-, German-, and Sweden-origin groups defoliated significantly later in autumn compared to Finland-origin group [25]. A similar explanation has been suggested in studies on different poplar hybrids [49–51].

In this experiment, trees experienced quite severe drought as the precipitation was only 17% of the long-term average. Trees of Finland origin were smaller, which means that their water demand is also lower and therefore they might be more resistant to severe drought [55, 56]. Southern origin groups had higher water demand due to bigger tree dimensions and they are therefore more susceptible to severe drought [57, 58]. Previous studies about deciduous tree plantations have shown that the competitively dominant trees are more sensitive to soil water supply than competitively smaller trees [10, 59]. On the other hand, lower *g*_day_ observed in the Latvia-, German-, and Sweden-origin groups, along with the absence of differences in *K*_shoot_ across all origin groups, indicates that genotypes from southern origins also possess the capacity to adapt to significantly diminished precipitation levels. Considering the increased drought-driven tree mortality in summers of Europe [60], the clonal selection for drought-sensitive soils should be done cautiously by mixing several genotypes to improve the general plantation resilience to climate change.

## Conclusion

In conclusion, the transfer of hybrid aspen genotypes from southern latitudes can significantly improve biomass production and CO_2_ fixation compared to currently commercially used genotypes in hemiboreal Estonia. We suggest that future hybrid aspen breeding programes do the final selection of the best genotypes at around the age of 10–15 years as we found that some geographic origins improved their growth ranking after the early growth stage. Northward transferred genotypes demonstrated lower net photosynthesis and daytime stomatal conductance and consequently, higher water use efficiency in drought conditions. The latter is a positive trait in drought conditions, which should be taken into account in the context of climate changes. While droughty conditions may obscure the direct relationship between *P*_n_ and growth, we posit that integrating physiological parameters alongside growth in genotype comparisons can give important information about their physiological adaptation and resilience. However, further studies are needed to describe genotypes responses to environmental factors comprehensively. We suggest a wider integration of genotypes physiological traits measurements to tree breeding and field-testing programmes to other tree species in the region.

### Electronic supplementary material

Below is the link to the electronic supplementary material.

Supplementary Material 1

## Data Availability

The datasets used and/or analysed during the current study are available from the corresponding author on reasonable request.

## Abbreviations

iWUE: intrinsic water–use efficiency
V: stem volume
H: height
DBH: diameter at breast height
M: stem mass
CAIM: current annual stem mass increment
RG: relative growth
*P*_n_: net photosynthesis
*g*_day_: daytime stomatal conductance
*E*: transpiration
*K*_shoot_: hydraulic conductance
N: nitrogen
