# Supplementary material for: Growth ranking of hybrid aspen genotypes and its linkage to leaf gas exchange
Source: BMC Plant Biol. 2024 May 22;24:435. doi: 10.1186/s12870-024-05104-6 (PMC11110358; doi:10.1186/s12870-024-05104-6)
Supplement: Supplementary file 1 — Supplementary Material 1 [file 12870_2024_5104_MOESM1_ESM.docx]

**Supplementary 1.**

**Table S1**. Source of the hybrid aspen genotypes (providers of the plant material and background information: Georg von Wühlisch (Germany), Guntis Grandans (Latvia), Anders Håkansson (Sweden), Lars-Göran Stener (Sweden) and Raimo Jaatinen (Finland)). The genetic specimens have not been deposited in a public herbarium.

| Clone origin country | Clone ID at trial | Clone ID | Origin | Average length of the vegetation period in days according to Lutter et al. (2016)*** |
| --- | --- | --- | --- | --- |
| Latvia | Lat1 | 4 | mother (*P. tremuloides*, 56.40`.34`` N) × father (*P. tremula*. 56.67 N) | 194 |
|  | Lat2 | 21 | mother (*P. tremuloides*, 56.67 N) × father (*P. tremula*, 56.10 N) | 189 |
|  | Lat3 | 23 | mother (*P. tremuloides*, 56.67 N) × father (*P. tremula*, 56.10 N) | 150 |
|  | Lat4 | 24 | mother (*P. tremuloides*, 56.67 N) × father (*P. tremula*, 56.10 N) | 193 |
|  | Lat5 | 25 | mother (*P. tremuloides*, 56.67 N) × father (*P. tremula*, 56.10 N) | 171 |
|  | Lat6 | 28 | mother (*P. tremuloides*, 56.67 N) × father (*P. tremula*, 56.68 N) | 150 |
|  | Lat7 | 41 | mother (*P. tremuloides*, 56.67 N) × father (*P. tremula*, 57.17 N) | 193 |
| Sweden | Swe1 | NF 1 | mother ( *P. tremula*, 55.99 N) × father (*P. tremuloides*, US ) | 190 |
|  | Swe2* | NF 7 | mother (*P. tremula*, 57.53 N) × father (*P. tremuloides*, 57.70 N) | 181 |
|  | Swe3 | NF 11 | mother (*P. tremula*, 57.50 N) × father (*P. tremuloides*) | 190 |
|  | Swe4 | NF 12 | mother (*P. tremula*, 57.50 N) × father (*P. tremuloides*) | 190 |
|  | Swe5** | NF 15 | mother (*P. tremula*, 55.89 N) × father (*P. tremuloides*) | 188 |
| Finland | Fin1 | C05-99-10 | mother (*P. tremula*, 60.22 N) × father (*P. tremuloides*, 45.17 N, Canada) | 165 |
|  | Fin2 | C05-99-16 | mother (*P. tremula*, 60.22 N) × father (*P. tremuloides*, 45.17 N, Canada) | 163 |
|  | Fin3 | C05-99-17 | mother (*P. tremula*, 60.22 N) × father (*P. tremuloides*, 45.17 N, Canada) | 162 |
|  | Fin4 | C05-99-34 | mother (*P. tremula*, 60.22 N) × father (*P. tremuloides*, 54.11 N, Canada) | 153 |
| Germany | Ger1 | W1 x Ihl 1 | mother (*P. tremula*, 52.27 N) × father (*P. tremuloides*) | 172 |
|  | Ger2 | W1 x Esch 9/85 | mother (*P. tremula*, 52.27 N) × (mother (*P. tremula*, 51.28 N) × father (*P. tremuloides*, 43.99 N, US) | 169 |
|  | Ger3 | W1 x Tur 141 | mother (*P. tremula*, 52.27 N) × father (*P. tremuloides*, 43.99 N, US) | 196 |
|  | Ger4 | Brauna 11 x Tur 141 | mother (*P. tremula*, 51.28 N) × father (*P. tremuloides*, 43.99 N, US) | 188 |
|  | Ger5 | W 95 x Tur 141 | mother (*P. tremula*, 52.27 N) × father (*P. tremuloides*, 43.99 N, US) | 191 |
|  | Ger6 | Ihl 3 x W 66 | mother (*P. tremuloides*) ×father (*P. tremula*, 52.27 N) | 179 |

**P. tremuloides* growing in local botanic garden

**Polish origin

***The length of the vegetation period is number of days from bud-burst (leaves emerge from the bud about 5 mm) to defoliation of all the leaves at age 6 years based on visual observations (Lutter et al., 2016).


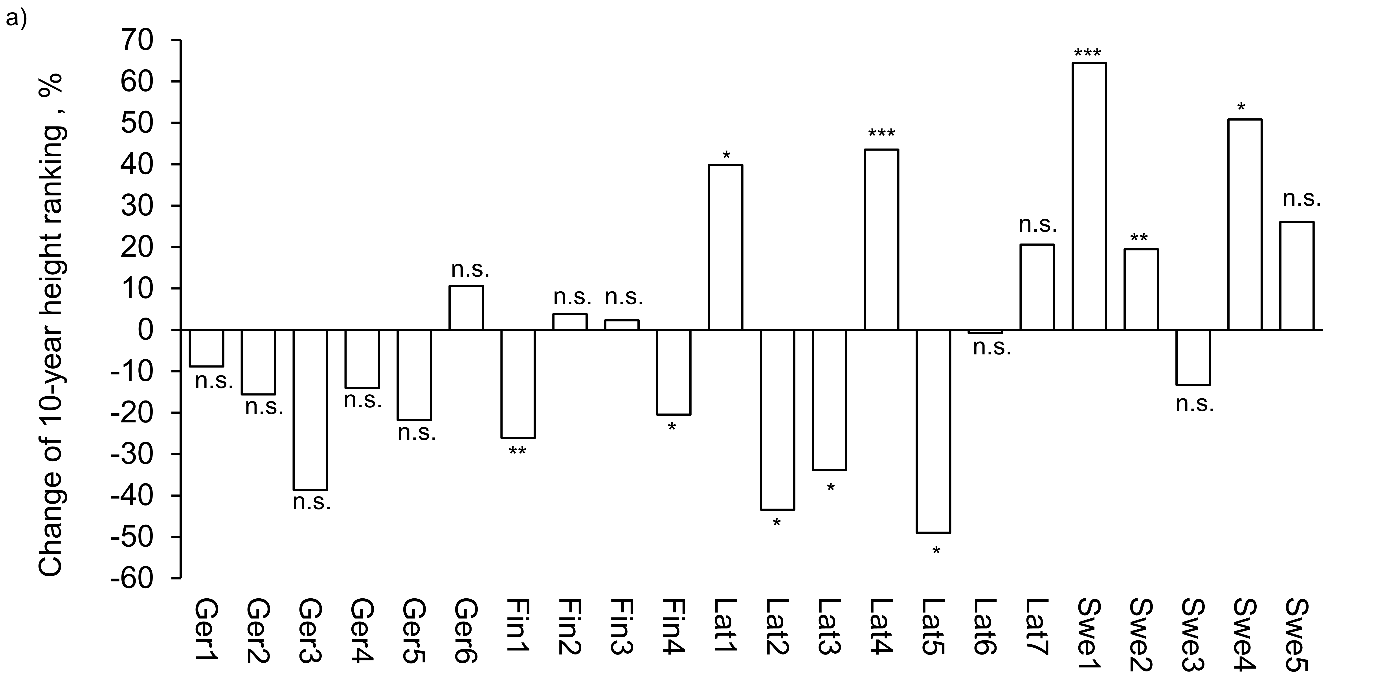


**Figure S1.** Ten-year (3- to 13-year-old) change of relative height ranking among the studied hybrid aspen genotypes. The significance of the mean change of ranking inside the genotype (i.e. difference from zero) is indicated as follows: *** P < 0.001, ** P < 0.01, * P < 0.05, n.s. - not significant.

**References**

Lutter R, Tullus A, Tullus T, Tullus H. Spring and autumn phenology of hybrid aspen (*Populus tremula* L. × *P. tremuloides* Michx.) genotypes of different geographic origin in hemiboreal Estonia. New Zealand Journal of Forestry Science, 2016;46:20. doi: 10.1186/s40490-016-0078-7
